# Listening to their voices: understanding rural women’s perceptions of good delivery care at the Mibilizi District Hospital in Rwanda

**DOI:** 10.1186/s12905-018-0530-3

**Published:** 2018-02-12

**Authors:** Zack Ndirima, Florian Neuhann, Claudia Beiersmann

**Affiliations:** Institute of Public Health, Ruprecht-Karls University Heidelberg, Im Neuenheimer Feld 324, 69120 Heidelberg, Germany

**Keywords:** Quality of care, Delivery care, Maternal satisfaction, Non-clinical aspects, Rwanda

## Abstract

**Background:**

Poor quality maternity care may lead to increased maternal dissatisfaction, and subsequent decreased utilization of health services or both. In a responsive health system, determining suitable delivery care, in the mother’s opinion, may lead to an improved quality of services and the mother’s satisfaction. In Rwanda, there is still limited knowledge and inadequate research regarding patient satisfaction and preferences, especially for women’s perceptions and needs during childbirth. This study captures rural women’s perception of good delivery care to understand aspects of care they consider important during childbirth.

**Methods:**

This qualitative study was conducted in the Mibilizi District Hospital catchment area located 350 km from the capital, Kigali, in the Western Province of Rwanda. It includes 25 in-depth interviews with purposively sampled rural mothers who had delivered in the hospital and five hospital midwives. Content analysis was performed manually.

**Results:**

With regard to interpersonal relations at the health facility, the women agreed on the need for respectful treatment in areas of sufficient privacy and had distinct preferences for the gender of the birth attendant, or husband’s presence during delivery. The women make a great effort to deliver in a health care facility and therefore, they expect to be assisted in a professional and safe manner. These expectations can be met on a personal level, but at times are counteracted by structural deficiencies and staff shortages.

**Conclusions:**

In gathering rural women’s perceptions of good delivery care, this study reveals what mothers in remote areas in Rwanda consider important during child birth. The women’s expectations, suggestions, and needs can enhance providers’ awareness of the women’s priorities during childbirth and serve as a guidepost for health services to increase the quality, acceptability and uptake of maternal health services.

**Electronic supplementary material:**

The online version of this article (10.1186/s12905-018-0530-3) contains supplementary material, which is available to authorized users.

## Background

Between 1990 and 2015 global maternal mortality decreased by 45% [1]. However, an estimated 800 women still die every day worldwide from pregnancy-related causes and most of these deaths occur in low- and middle-income countries [1]. Sub-Saharan Africa is the region with the highest maternal mortality ratio (MMR) [1]. Yet, some African countries have achieved remarkable progress in achieving the Millennium Development Goal 5 (MDG5: Improve maternal health) and its related target of reducing MMR. Rwanda has almost met the 50% reduction target since MMR decreased from 476 (in 2010) to 290 (in 2015) [2]. Rwanda is among several countries that have recorded a high relative reduction in maternal mortality [3].

A sub-Saharan low-income country whose health system was once paralyzed by the 1994 genocide, Rwanda is commended for significant progress in maternal health, and especially for improved access to health services. Even so, its health system is not spared from the aforementioned challenges facing health care in resource-limited settings. These inadequacies affect the quality of maternal care and in particular intra-partum and delivery care since this is the time period that represents the highest number of maternal deaths [4]. In Rwanda, there is limited knowledge and research on non-clinical aspects of delivery care. Knowledge of the mother’s perception of her delivery experience and her non-clinical delivery needs may be instrumental in developing contextual and culturally sensitive strategies of delivery care and improving its quality [5]. Moreover, such knowledge may foster good practices leading to improved quality of services and subsequent mother satisfaction.

This study focuses on quality of care during labor and childbirth and probes for women’s opinions and perceptions on the care they receive during this process. The focus is to understand the non-clinical aspects of care that rural woman in Rwanda consider important during childbirth.

## Methods

This qualitative study was performed as a requirement of an MSc in International Health thesis by the first author (a male physician [MD]) in May and June 2014, in one district hospital in a rural Rwandan setting. The study included 20 in-depth interviews (IDIs) with mothers who had given birth within ten weeks prior to study start and five IDIs with midwives present during the same time period.

### Study area

The study was conducted in Mibilizi District Hospital, which is one of 39 district hospitals in Rwanda. Situated in the Western Province of rural Rwanda about 350 km from the capital, Kigali, the facility is staffed by general practitioner doctors. The hospital provides services to in-patients and outpatients on referral from health centers and serves as a gateway to more specialized care from specialist doctors in referral hospitals. The hospital offers comprehensive emergency obstetric care [6] on a 24-h basis. The 65 maternity-bed unit covers a catchment area of approximately 226,000 inhabitants [7]. Women who frequent the Mibilizi maternity unit are mostly rural mothers with low social-economic status and a low level of formal education. Most women are covered by the community-based health insurance (CHI) ‘*Mutuelles de Santé’.* The hospital suffers from common problems hospitals in resource-limited settings such as inadequate equipment and staffing problems [7].

Over recent decades, the Rwandan government has introduced specific health system developments, e.g. a countrywide independent CHI scheme in 1999, and a performance-based pay initiative in 2005 [8]. The CHI has improved utilization of maternal health services and skilled-birth attendant utilization [9–11]. Provider performance-based payments have led to increased use and quality of several crucial maternal services, for instance, obstetric care [12]. The CHI is a three-tiered premium system based on ‘Ubudehe’, a socioeconomic hierarchical system that exempts the poorest from obstetric fees and subsidizes the least poor [13]. In addition, as encouragement for institutional delivery, services are free for women who complete four antenatal care visits (ANCs) [14]. Otherwise, an institutional delivery is $3.22 for a woman with health insurance versus $4.83 for a woman without health insurance [7].

### Study participants

Women who had delivered in Mibilizi District Hospital within ten weeks prior to start of the study were purposively sampled to represent typical cases via maternity registers [15]. Ten primiparae (three cesarean sections and seven spontaneous deliveries) and ten multiparae (three cesarean sections and seven spontaneous deliveries) were included. The mothers were contacted through the child immunization appointment register (appointments at six and ten weeks for diphtheria and polio vaccines) prior to their vaccination appointment through the community health workers for information and consent for study participation. Sixteen of the IDIs were held at a youth center away from the health facility that was considered a neutral place. Four IDIs were held at the participants’ home for failure to show up at the planned interview venue. There was no third party in the room during the interviews at the youth center. The interviews at home where inevitably carried out in the presence of family members.

Midwives of both sexes with at least six months of working experience at Mibilizi District Hospital were selected for IDIs. Additional eligibility criteria were that they had not been on leave for two months prior to start of study, and worked both night and day shifts. Interviews with midwives were held at the maternity department during their free time.

### Study instruments

The interview guide focused on 12 thematic areas developed a priori from a literature review of studies on the quality of maternal health and satisfaction in developing countries, distance and transport to health facility, decision to seek care, preference of place of delivery, health provider empathy, communication, promptness of care, birth companion/husband’s presence, birth attendant preference, privacy, cost of services, availability of information, and hygiene and cleanliness of place of birth (Additional files 1, 2 and 3).

The interview guide was translated to Kinyarwanda (local language) by the first author (ZN). It was not piloted, but rather cross-checked by and discussed with the head midwife of the study site, before and during the course of the interviews.

### Data collection, management and analysis

ZN conducted the in-depth interviews in Kinyarwanda, taking notes and voice recordings. Prior to each interview, the researcher introduced himself and asked for written informed consent which was given in all cases. IDIs lasted about an hour and a relationship with the study participants was established via informal talk before commencement. Transcription (in Kinyarwanda) and subsequent translation to English were done on a daily basis by ZN. Transcripts were not handed back to participants for comment and/or correction. Field notes originally taken in Kinyarwanda were translated to English, examined and then added to the text subject to analysis. Data was manually analyzed by ZN applying content analysis. For coding, a complementary approach of deductive and inductive coding was applied. Principal thematic areas were developed from the interview guide and literature. Categories and sub-categories were formed in line with the major themes and applied to the data (deductive approach). In parallel, while reading the data, categories, and sub-categories emerged that had not been previously identified, so additional codes were added (inductive approach) (Additional files 4 and 5).

### Ethical consideration

Ethical approval was obtained from the Ethikkommission, Medizinische Fakultät Heidelberg with reference number ***S-153/2014*** and from the National Health Research Committee, Rwanda with reference number ***NHRC/2014/PROT/0152***. Prior to each interview, written informed consent in Kinyarwanda (local language) was obtained from the participants including consent for voice recordings. In cases of illiteracy, the participant was informed orally and consented with a fingerprint. Study participants had the right to withdraw from the study at any stage. Pseudonymization and confidentiality was guaranteed.

## Results

The results of this study provide a detailed description of the participants and their opinions on the following themes: decision to seek care and preference of place of delivery, experience during the birthing process, health provider empathy, presence of husbands and emotional support, birth attendant preference and privacy, availability and sharing of health information, availability of drugs, supplies, and equipment, hygiene issues, promptness of care, distance and transport to the health facility, and costs of services.

### Detailed description of study participants

As planned, all women interviewed were from the catchment area of Mibilizi district hospital and had delivered at the Mibilizi District Hospital; ten were primiparous and ten multiparous, and 14 had had spontaneous vaginal deliveries and 6 had had cesarean sections. They lived within 5–30 km from the facility. The women were mainly from a low socio-economic class. A number of the women were subsistence farmers and their husbands were, for example, occupational laborers, carpenters, fishermen, teachers, or military. Most of the women were semi-literate with few years of primary school education. Among the 20 women, only four had been to secondary school. Their husbands had similar levels of education. The women’s ages ranged from 18 to 43 years, and they had between one and ten children. Their (most) recent delivery had occurred within ten weeks prior to the study. Most women were married (marriage between 15 and 26 years of age) and three had had deliveries out of wedlock. Sixteen of the 20 women had CHI.

Among the midwives interviewed, four were female and one male. Three had three years of midwifery training and were certified midwives, whereas two had studied nursing in secondary school. They all had experience in the maternity unit that ranged from two to nine years. The midwives were between 24 and 48 years of age.

### Decision to seek care and preference of place of delivery

The women indicated that their decision to seek care was influenced by family opinion, previous delivery experiences, fear of complications, community health worker mobilization, desire to shorten labor duration (receiving labor inducing and hastening drugs), common practices, hygiene, affordable cost of services and the rare need for instrumental delivery (cesarean section).

Midwives alluded to the women’s impression that community health workers are of paramount importance in mobilizing and sensitizing women to consider institutional deliveries and close follow up during their pregnancies.

*“The community health workers sensitize us to attend ANC*^1^, *you can never know how they detect that you are pregnant….as soon as you are 2 months pregnant they are at your door without your notice!” (mother 09 multiparous).*

*“At hospital they manage the complications better because they have gone to school and in case they fail they can refer you but at home you can lose your life easily.” (mother 04 multiparous).*

In some cases women came to deliver at the hospital because their previous deliveries had been cesarean sections, whilst others sought for care after delivery failures at home by traditional birth attendants.

### Women’s birth experiences

Women had ambiguous feelings about childbirth and considered it to be a state between life and death.

*“I can give birth again but not soon…eeh, I felt like I was dying. I would like to forget that experience a little” (mother 03 primiparous).*

*“Giving birth is painful, I wouldn’t wish to do it again but the joy in it is the baby. You basically bring someone to earth” (mother 06 multiparous).*

On one hand, in the event of success, a birth brought happiness and respect from the community. On the other hand, it evoked feelings of failure, regret, desperation, self-blame, and naiveté when there were negative outcomes like stillbirths and cesarean sections.

Midwives reported a feeling of satisfaction, pride and encouragement whenever mothers left the hospital with healthy babies. However, they confessed dissatisfaction and helplessness in cases of a poor outcome attributed to reasons under or out of their control.

### Health provider empathy, presence of husbands and emotional support

The mothers stressed that health providers’ good attitudes towards them during labor raised their self-esteem and confidence. Hence, they condoned friendly, respectful attitudes by the midwives and resented abusive and degrading language. In general, they commended birth attendants for being welcoming and warm despite a few exceptions.

*“…you know labor pains are so painful, I had the feeling I had become so demanding and at times didn’t follow their instructions […] but it wasn’t me. Despite all this, they kept their calm, remained caring and reassured me that I will give birth” (mother 10 primiparous).*

*“Upon arrival the midwife examined me and told me that I am at 2 centimeters (cervical dilation). I was not seen for 4 hours until I asked to be examined for my progress but she said, ‘keep moving around the labor room you are not yet complete.’ I felt somehow abandoned and I feared for my unborn baby’s safety so I had to scream loud to attract attention. She told me to keep silent because that was disgracing and immoral of a lady” (mother 03 primiparous).*

Out of the 20 respondents, five had been unaccompanied by their husbands/partners, four husbands/partners had been present in the delivery room, and 11 husbands/partners had been present on the hospital premises only. Women indicated that their husbands’ presence during delivery was important for their emotional and physical support as well as decision-making purposes and logistical support. It was noted that husbands might help in activities like buying: drugs from the pharmacy, food, baby necessities, and etcetera. Women felt protected and secure in their husbands’ presence, and thus relaxed to take on the birthing task.

*“My husband’s help felt like scratching that itch I couldn’t reach myself! The hospital pharmacy didn’t have a particular drug, he ran and bought it from a pharmacy outside. It was good they allowed him to be near me” (mother 02 multiparous).*

*“I find my husband’s presence important because he assists in care and I feel protected. He is my immediate advisor so he must be there. We share the burden” (mother 13 primiparous).*

*“My husband had to be there; that is why we married, if he didn’t wish to be there then I would have reconsidered our love” (mother 20 multiparous).*

However, some mothers, especially those who were multiparous, expressed a preference for their mothers-in-law over their husbands because a mother-in-law could nurse them better and these women considered delivery to be a feminine act. The women feared their husbands would become less sexually attracted to them, if they saw them giving birth.

*“Well, my husband should be there but outside…really he doesn’t want to see what happens down there…it might cut his appetite and besides my mother-in-law can nurse me better. My man cannot know women’s stuff” (mother 20 multiparous).*

Midwives emphasized the significance of husbands’ presence for making critical decisions like, surgical sterilization, cesarean section or consent for procedures like blood transfusion, hysterectomy, and etcetera.

*“I had had three caesarean sections and was having my fourth. After delivery the doctor asked if he could tie my tubes…I wished to but I was hesitant to take the decision on my own in my husband’s absence” (mother 18 multiparous).*

*“[…] bleeding heavily she was unconscious, we needed consent for blood transfusion but we were in a dilemma because her faith prohibited it. We had to consult the husband for consent…he approved and signed…that put us on a safe side” (midwife 03).*

### Birth attendant preference and privacy

All the women in this study stressed the need for giving birth in a secluded place. They found it embarrassing to deliver in an open place where their private parts were exposed to strangers. Women indicated that privacy was respected in this hospital, but not always. They pointed out that even with home births they delivered in private and in the absence of males, therefore, they expected the hospital to respect the need for privacy during childbirth.

*“…they hurriedly moved me on a stretcher from the labor ward to theater through corridors with many patients and I think caregivers, my breasts exposed…. I felt uncomfortable but it was an emergency”(mother 14 primiparous).*

Regarding attendant preference, most of the women acknowledged that attendants were insufficient in number; hence, they had little possibility of choosing a birth attendant. They expressed trust in attendants’ competence except for interns. On one hand, some women preferred to be birthed by males assuming they knew more, whereas some preferred females for reasons of privacy. Midwives concurred with the women that some women prefer male attendants because of the assumption that they are doctors and therefore know more.

*“On my previous delivery I was delivered by a female attendant but personally I prefer male attendants because they are more empathetic and seem to know better; I know, because they have delivered me in the past” (mother 09 multiparous).*

*“I was delivered by both male and female but I would prefer a female to deliver me; she is a woman too, she knows exactly what you are going through. She knows how labor pains feel” (mother 04 multiparous).*

### Information sharing

Most women expressed the need for information about what was happening to them during and after delivery and about their baby’s health. A number of women reported they had not been informed or educated during either their hospital stay or upon discharge. All the primiparous mothers expressed the need to be educated on vaccination, family planning, nursing, and wound healing.

*“I told them I had applied hot water…they scolded me saying my sutures were broken because of the hot water hence healing was problematic. I blame it on the nurses, they never told me how to nurse the wound” (mother 07 primiparous).*

Multiparous mothers said they were less likely to receive education with the assumption that they were experienced, but every delivery was unique, so they expressed need for guidance too.

On one hand, midwives explained that upon discharge women were educated on breastfeeding, nutrition, vaccination, wound care and hygiene. On the other hand, they admitted that some women leave the hospital without counseling or health education due to the work overload.

*“Our zone is highly reproductive and the illiteracy rates are high…..when we have time we do counsel the women on family planning. Unfortunately we don’t do it routinely because of time yet it is needed” (midwife 02).*

It was reported that complications after discharge were common in mothers with low levels of formal education. Poor living conditions and inadequate nutrition were assumed to be the reasons the women could not comply with given instructions.

*“...they are poor and less educated….it is hard for them to maintain hygiene at home….hence they come back with infected wounds” (midwife 02).*

Furthermore, some women mentioned that it was important to be educated/informed as a couple (husband/wife) for reasons of reminiscence and shared compliance.

*“…the nurses warned me not have sexual intercourse in at least four weeks….but on the third week my husband wanted me…I tried to reject but he forced it….it took so long to heal because of this. I believe he would have understood had he been there to listen to the nurses’ advice” (mother 03 primiparous).*

### Availability of drugs, supplies and equipment

It was reported that drugs and supplies were generally available, but some particular supplies/drugs were occasionally out-of-stock since demand exceeded supply. Blood transfusion products were said to be hard to access; they are ordered from a blood bank far away that is sometimes short of blood for particular blood groups. Oxygen was another element that the women reported to be inadequate. The number of oxygen cylinders is disproportional to the beds in the labor room. In addition, midwives indicated a need for new and up-to-date labor monitoring equipment that might help expedite services and improve quality of care. Some midwives expressed being demoralized and disappointed about the shortage of material to perform in critical situations. They felt useless and frustrated being unable to help patients in critical conditions they knew well how to manage.

*“It is risky to operate in such a condition imagine we have only two oxygen cylinders while the delivery tables are six” (midwife 03).*

*“…she bleeds, her condition worsening by the minute, as you look on helplessly, but what do you do…you get her referred to the tertiary hospital and pray she survives… The problem we have is the blood bank is far away in Karongi district” (midwife 02).*

### Hygiene issues

In general, the hygiene in the labor rooms was reported to be adequate with clean rest rooms and constant running water. However, admission wards were said to be filthy and less ventilated since they were overcrowded with patients.

*“…labor room was clean and ventilated but after delivery I was transferred to a crowded room that was filthy and rowdy. I couldn’t wait to be discharged the following day” (mother 13 primiparous).*

Midwives were content with the cleanliness in the delivery rooms and with the stringent infection control teams at the facility. However, they pointed out that some buildings are in poor condition and not well adapted for maternity and labor rooms, delivery tables. An inadequate number of beds has led to patient overcrowding. They were concerned that this affects the quality of services and may increase the risk of nosocomial infections.

*“..we have 58 beds in the admission ward but have 71 patients at the moment; some women have to share a bed” (midwife 03).*

*“…post-operational infections here are partly due to the overcrowding….we mix the patients because we lack space” (midwife 05).*

### Promptness of care

Furthermore, almost all of the women agreed there was less waiting time upon arrival at the emergency compared to the past, but concurred that follow up after admission was limited. They reported that they were given attention mostly on arrival and when they neared birth.

Multiparous women who had delivered in the same facility earlier noted an improvement in promptness of services.

*“When I arrived I was complete, they delivered me immediately but had some bleeding of which they stopped. One hour later, I went to the bathroom and fainted from there, they promptly attended to me, gave me IV fluids and did some suturing down there. If it had been before I would not have survived. Services were so poor and slow here before” (mother 08 multiparous).*

Midwives in the Mibilizi maternity unit described their work as overwhelming in the face of inadequate staff at the facility. They noted that often they have to exceed the normal working 45 h per week and this is even worse when colleagues take leave or attend conferences. They expressed the desire to serve at their best, but are held back by insufficiencies out of their control.

*“Sometimes we burnout from work….in such cases patients fall victim. We tend to be intolerant, inpatient and tempestuous…may even use inappropriate language toward patients unintentionally” (midwife 02).*

### Distance to the health facility and availability of transport

The women reported to have travelled for 1–5 h by foot to reach the health facility for delivery. Some of the women were carried to hospital by their kin on “Ingobyi” (a traditional stretcher), bicycles or motorcycles. Mibilizi district is known for its heavy rainfall, hilly landscape and underdeveloped road infrastructure, hence the problematic use of motorized transport.

*“…..we travelled by foot for an hour accompanied by his young brother, of course with many breaks but I got tired on the way…I felt like the ‘waters had broken’ (rupture of membranes), was suddenly not breathing anymore just laid on the ground and thought oh this is my last day of life. He called a friend who arranged for a motorcycle that reached us in about 45 minutes…..when I got to hospital I gave birth an hour after arrival, God is great” (mother 04 multiparous).*

The midwives concurred with the women on the problematic physical access to the hospital. They mentioned that pregnant women had to walk for long distances on difficult terrain and sometimes in bad weather because the number of available ambulances does not meet the demand.

*“Some patients trek from Nyabitibo near the Burundian border, about 6 hours by foot, up and down hill. We have such a wide catchment area and a difficult terrain” (midwife 03).*

*“Sometimes the community health workers call for an ambulance while none is available because of other emergencies… such situations paralyze us” (midwife 01).*

### Cost of services

The cost of services was reported to be fair compared to the services provided. However, patients not able to pay at all can be exempted from costs in accordance to a stratified premium system in effect since 2012. In addition, as encouragement for institutional delivery, services are free for women who complete their four ANCs.

*“We keep them in hospital for some days until they are able to pay, some sell their possessions or are exempted in case they belong to the abject poverty strata per the national wealth household categorization” (midwife 02).*

*“After delivery I was told not to pay a penny because I had attended four ANCs and delivered normally….I expected it because I was informed during the ANCs” (mother 09 multiparous).*

Midwives found the pay for services extremely low. They reported a remarkable increase in services uptake related to the introduction of CHI sometimes leading to work overload or insufficient material resources.

## Discussion

In traditional societies, childbirth is viewed as a normal event in the life of a woman despite the increasing medicalization of the delivery process [16]. Childbirth may therefore take place at home, but there are also factors that lead women to deliver from health facilities. In Rwanda there is an increased uptake of maternity services, in particular, ANCs and hospital deliveries [12]. However, knowledge of women’s perception of delivery care in rural Rwanda is still scarce.

Listening to the voices of the women of the rural low social class in this study reveals important aspects for improving the quality of care and responding to their needs and expectations. Important considerations during the process of childbirth and post-partum for these women included: privacy, emotional support from both provider and family, presence of birth companion especially husbands, provider empathy, promptness of care, proximity, adequate infrastructure and cleanliness of the facility, low cost of services and information during labor and the postpartum period on themes like wound care, nutrition, vaccination, family planning and breastfeeding.

In as much as there is significant focus on the improvement of the quality of clinical medical services in developing countries, there is an equal need to emphasize non-clinical components of delivery care. In the current study, women often referred to giving birth as a perilous act—a state between life and death—and one that evokes feelings of regret, desperation and self-inflict. Therefore, there is an expressed need for psychological and emotional support, privacy, and respect, as well as accessible and affordable services. These aspects of maternity care were shown to be influential on women’s expectations in other studies, which in turn influenced acceptability, service uptake and women’s satisfaction [17].

A positive attitude by attendants towards pregnant women and psychological confidence in the relationship between the woman and the midwife is important for the emotional aspects related to childbirth [18]. One of the main factors causing a negative birth experience is a midwife who did not listen to the needs of the woman and her partner [19]. In this study, non-abusive language, respect, empathy, encouraged the woman and raised her self-esteem and confidence and might have had positive effects on the process of labor. Women’s feelings of support and good quality care were also invoked in this study through factors such as less waiting time and prompt and continuous care from the midwives throughout their hospital stay. A short waiting time is an aspect that may indicate good service provision, reassure the woman and raise her expectation of subsequent good quality of care. This confirms the narratives of distressed women in a South African study that reported women felt neglected and abandoned in their first stage of labor, in particular [20]. A review of randomized control trials in developing and developed countries indicated that continuous intra-partum support is likely to lead to a shortened labor, and a normal birth and reduces the likelihood of dissatisfaction [21]. Inadequate and insufficient material and human resources, i.e. inadequate numbers of birth attendants, few delivery tables and ambulances, and a lack of drugs/blood products and resuscitation fluids, were reasons given by midwives in this study for delays and inadequate care. This finding is supported by other studies from rural health facilities in Tanzania [22, 23]. Access and availability of oxygen and blood products was identified in particular as a major challenge in the hospital in this study. Reportedly, the blood bank is very far from the hospital. It is recommended that concerned health authorities review the supply chain of the above products.

The presence of family members is another key aspect that the women in this study believed constituted good care and this is reflected in literature from India [24]. Husbands/partners play an important role in supporting the women physically and emotionally, and also in decision-making or logistical support. This was underlined also by the midwives in this study who stated that birth companions are “a birth attendant’s aide” i.e. they help with nursing, counseling and provision of necessities, which may include purchases outside the hospital. In particular, the husband’s presence was reported to boost the woman’s confidence, arouse a feeling of support and deter feelings of abandonment and might thus contribute to a delivery without complications. A husband’s presence and commitment is also necessary for education by the health provider on postpartum care and decisions on family planning. In addition, some medical procedures like surgical sterilization, blood transfusion and cesarean section were reported to require both the woman and husband to consent. This is interesting, since traditionally in Rwanda men are forbidden from witnessing the act of birth, because it is believed to affect the couple’s relationship (e.g. the man losing interest in his spouse). However, cultural transformation in this community has unveiled a range of benefits as expressed by some (not all) the women in this study. While currently, male partner involvement in maternal health still appears to be low in most of sub-Saharan African countries, there seems to be a shift towards father’s participation also reported from other countries in the region, Nigeria and Uganda [25, 26]. Other than the husband and/or a male attendant being present during the delivery, the women expressed their desire for privacy and a secluded place for examination and delivery, as other studies have shown [24, 27]. However, this wish is not always respected. Maternal services devoid of privacy reflect disrespect for the women and create the impression that they have lost control of the situation. At the hospital in this study, some women were examined on their beds in the presence of third parties in the event of an overflow of women in labor. However, the lack of resources such as labor and examination rooms and human resources make adequate privacy not easy for the provider to ensure at all times. Women in the current study also expressed discomfort with some procedures especially when performed by male attendants or multiple individuals. Some women had a preference for female attendants because of traditional or religious beliefs, whereas others preferred male attendants thinking they knew more than female attendants and were emotionally stronger to help in complicated situations [16]. However, in developing countries where human resources are scarce, the preference for female attendants for privacy is often not a priority.

Imparting information to women during labor and the postpartum period is another component of maternity care deemed extremely important. A literature review indicated that information imparted during labor might evoke a feeling of control and competence in the process of normal birth by implying the likelihood of delivering without complications [21]. Findings of our study concur with the statement that denial of information during labor reduces women to states of passivity in which they become unable to actively participate in their own birth experiences [20]. In the current study, the lack of information during the labor and postpartum periods consequently evoked anxiety and helplessness. Women expressed a need to be notified on their progress each time they were examined, which concurs with findings in Ghana [17]. For example, the women wanted information on the extent of cervical dilation and needed education on family planning, vaccination, breastfeeding and hygiene. Clear impartation and subsequent understanding of information on self-hygiene and nursing may reduce risks of postpartum infection [28]. Unfortunately, there is less impartation of information to mothers in rural and developing settings, and South Africa is an example [20]. Social determinants of health such as living standards and education level, which are mostly poor in rural African settings, may be linked and add to the burden of postpartum infections. This is in line with findings of our study where midwives referred to the high illiteracy levels in the facility’s catchment area. They pointed out that it is difficult for mothers from a low social economic background to ensure maximum hygiene and to avoid wound infections during the postpartum period given their poor living standards. Yet, it could also be possible that the women with complications in wound healing had already acquired an infection in the hospital. Both the women and the midwives saw overcrowding as a threat to cleanliness and hygiene.

To address the need to receive and impart information during the labor and postpartum period, it is recommended to strengthen Information Education Communication services. There is need to inform and explain the progress of a woman’s labor and educate them during the postpartum period on topics like breastfeeding, wound care, nutrition, family planning and vaccination. Men should be included in this sensitization. Since a husband’s presence during delivery is important for the laboring mother and/or facilitates the care provider at the same time, it is recommended to sensitize the men to accompany their wives during labor. Particular information could be given on how to assist their women when accompanying their wives during labor. Sensitizations may be done at a community level.

Cost of services is another factor influencing mothers decision where to seek care [24]. The women in the current study regarded their delivery costs as fair. Most of the women interviewed were members of the CHI scheme that gives reduced prices. Yet, midwives in the current study see a disproportion between the services provided and the cost of services. They find the cost to be extremely low and assert that the increasing service uptake affects the quality of services provided in a negative way. The increasing service uptake aggravates the already existing lack of sufficient skilled birth attendants. The Ministry of Health has tried to curb this by establishing a workforce-building strategy through its *Human Resources for Health* program and performance-based incentive scheme [4]. Studies from other African countries indicate that a reduction of user fees can be effective in favoring equitable access across socio-economic groups and health insurance increases the likelihood of hospital deliveries [29–31]. However, rising hospital deliveries in developing countries may exceed the health systems capacity in terms of infrastructure, material and human resources [32].

### Study strengths and limitations

The study has several strengths and limitations. Strengths of the selected sample were that respondents were of various parities and the mode of delivery differed, thus increasing the variety of phenomena and perception. This increased the credibility of the study. In addition, triangulation of the women’s interviews was done with midwife interviews to determine where ideas/opinions concurred or differed. To minimize recall bias, respondents were eligible if they had delivered within ten weeks prior to study. Instead of holding focus group discussions, in-depth interviews were carried out to ensure confidentiality, comfort and to avoid competitiveness. In addition, the first author is a native speaker of the local language and conducted all the interviews himself, making training of external interviewers unnecessary.

The first author is a male, which can have a negative effect when interviewing women in a paternalistic society. He had worked in the maternal health field implying that personal biases may not be ruled out. The study was carried out in one district hospital, which does not reflect the picture in other parts of Rwanda. Furthermore, all birth outcomes in this study were live births. Inclusion of mothers who had had stillbirths might have covered a wider variety of respondents, but this was not feasible. The approach used to contact the study participants (through the vaccination unit) could have influenced the respondents’ opinions for reasons of courtesy. To mitigate this, interviews were held in a youth center away from a hospital and in the absence of health care providers. Lastly, the interview guide employed in study was tested because of time constraints. To customize it, adjustments were made after consultations and discussions with the head midwife, and initial interviews. Results were not reported back to the participants, but will be available to a wide audience through this publication.

## Conclusions

The study presented here diverts the focus of childbirth circumstances to the women receiving the care and the inclusion of non-clinical aspects of care germane to maternal health, through listening to their voices. In gathering the rural woman’s perception of good delivery care, this study reveals what mothers of low socio-economic status with low education levels in remote areas of Rwanda consider important during child birth. It highlights the necessity of health providers listening to the woman’s voice about her needs and expectations in the bid to have the services she receives improved. The suggestions raised by the women are meaningful and part of what is considered good quality of care. They also show that maternity services can and should provide options/choices e.g. with regards to the presence of husbands and their role or offering family planning counseling on demand. Some of the women’s recommendations can be implemented with almost no additional direct cost. On the other hand, it is evident that Rwandan health systems and services need strengthening to reach and maintain levels of quality. The aforementioned expectations and needs of the women are expected to enhance awareness on the provider’s side about what the women considered to be prime during childbirth. If these expectations are met, there is the likelihood that the acceptability and uptake of maternal health services in Rwanda will increase.

## Additional files

Additional file 1: Women interview guide in English. (PDF 59 kb)

Additional file 2: Women interview guide in Kinyarwanda. (PDF 49 kb)

Additional file 3: Midwive’s interview guide. (PDF 67 kb)

Additional file 4: Coding scheme (illustrating interview text analysis). (PDF 82 kb)

Additional file 5: Table COREQ checklist (consolidated criteria for reporting qualitative studies 32-item checklist). (DOCX 20 kb)

## Abbreviations

ANC: Antenatal care
CHI: Community health insurance ‘*Mutuelles de Santé*’
IDIs: In-depth interviews
MMR: Maternal mortality ratio

## Footnotes

1 Women referred to ANC generally as an examination during pregnancy. We replaced “examination” with “ANC” in the transcripts.
