# Supplementary material for: Listening to their voices: understanding rural women’s perceptions of good delivery care at the Mibilizi District Hospital in Rwanda
Source: BMC Womens Health. 2018 Feb 12;18:38. doi: 10.1186/s12905-018-0530-3 (PMC5809806; doi:10.1186/s12905-018-0530-3)
Supplement: Supplementary file 1 — Women interview guide in English. (PDF 59 kb) [file 12905_2018_530_MOESM1_ESM.pdf]

WOMEN INTERVIEW GUIDE

Interviewer	Name:		Date:		Location:
Interviewee	Name:		Date of Delivery:		Mode of Delivery:
	Age:	Parity:	Marital status:	Education:	Economic activity:

Questions

1. Please tell me how the recent delivery of your baby/ babies took place.

2. Could you tell me how you went to hospital for delivery?

   Probes:
   - Distance from home to health facility
   - Transportation from home to facility
   - Weather and terrain
   - What costs did you incur in this process?

3. How did you decide to go to the hospital for delivery?
   Probes:
   - Who decided that you go to the hospital?
   - Why wasn't a home delivery an option?

4. What are the services you received at the hospital?
   - Pre-labor counseling (information)
   - Ultrasound
   - Blood tests
   - Clinical examination
   - Labor management
   - Post delivery care
   - New-born care

5. How did you perceive the services you received?

   Probes:
   - Interpersonal behavior of the providers (kind, compassionate)
   - Perception of negligent care
   - Promptness of care in case of complication, laboratory results and procedures.
   - Pain management

6. How long did it take to be attended to after arrival at the hospital?
   Probe:
   - What is your opinion on the waiting time?
     What was your perception on the reception

7. Did you pay for any service during delivery?

   Probes:
   - What did you pay for?
   - What was the cost?
   - What was your perception on the cost?
   - How did it feel not to pay for the services?

8. What sex was your birth attendant?
   - Were you comfortable with her/him?
   - Privacy & confidentiality
   - How much faith/trust did you have in her/him?

9. How often were you examined before delivery?

10. Tell me how the contractions felt like.
    Probes:
    - What kind of care did you receive during this duration?

11. How was the process of expelling the baby "pushing"?
    Probes:
    - How long did it take?
    - Were you assisted?
    - Perception of midwife's attitude during this period.
    - Did episiotomy? If yes: Was it painful?

12. How long did it take to deliver your placenta?
    Probes:
    - What is your perception on the time duration?

	13. When did you first see your baby?<br>Probes:<br>- Was it close to you?<br>- Did you breastfeed immediately?<br>- When were you told its sex?<br>14. Did the baby receive any kind of care immediately after delivery?<br>What kind of care?<br>Probes:<br>- Perception of care<br>- What kind of care?<br>- Baby clothes available immediately/ baby warm?<br>- Umbilical cord was ligated/tied on spot<br>- If no care, how did you feel about it?<br>15. Deliveries out of the hospital may include some traditional rituals that you might not get in hospital, how important are these rituals to you?<br><br>16. Tell me about the caregiver's visits during your stay in the post delivery ward?<br>Probes:<br>- How often?<br>- Pain managed<br>- Baby examined<br>- Advice on breastfeeding and personal hygiene<br>17. Did you receive any information upon discharge?<br>Probes:<br>- What kind of information?<br>- Is the information helpful?
Closing	I thank you for sharing experience with me and giving me your precious time. Are there any questions or further experience you would like to share with me?<br>May I contact you in case I need further information? In case you want to contact me or you have any questions please don't hesitate: Tel….<br>I
