# Supplementary material for: Listening to their voices: understanding rural women’s perceptions of good delivery care at the Mibilizi District Hospital in Rwanda
Source: BMC Womens Health. 2018 Feb 12;18:38. doi: 10.1186/s12905-018-0530-3 (PMC5809806; doi:10.1186/s12905-018-0530-3)
Supplement: Supplementary file 2 — Women interview guide in Kinyarwanda. (PDF 49 kb) [file 12905_2018_530_MOESM2_ESM.pdf]

IBIBAZO KUBABYEYI BA BYARIYE MU BITARO BY'AKARERE KA MIBILIZI

Ubaza	Amazina	Itariki	Ahantu

Ubazwa	Amazina	Igihe yabyariye	Uburyo yabyaye	Imyaka	Imbyaro	Akazi	Amashuri	Urubatse

Ingingo	Ikibazo	Imfasha mibarize
	Wambwira uko kubyara byagenza vubahano?	
	Wajiye gute kubitaro?	- Indeshyo y'urugendo<br>- Uburyo<br>- Ikirere n'imiterere y'inzira<br>- Igiciro cy'urugendo
	Ninde wafashe icyemezo ko ujya kubitaro?	- Kuki kubyariri imuhira bitashobokaga
	Ni izihe serivisi wahawe ujezeyo?	- Isuzumwa mbere yo kubyara
	Serivisi wahawe wa zibonye ute?	- Imivugire yabagaganga/abaforomo<br>- Kwitabwaho<br>- Kubanguka mukwitabwaho<br>- Kugabanyirizwa ubu babare
	Wamaze igihe kingana gute kugirango wakirwe?	- Wategereje igihe kinga gute mbere yuko wakirwa<br>- Ubitekerezho iki
	Ese hari serivisi wishuriye?	- Iki<br>- Angahe<br>- Birishurika<br>- Kutishura byafashije iki
	Wakiriwe n'umuntu wikihe gitsina?	- Wari wisanzuye<br>- Wafashwe bikwiye<br>- Ikizere kuwagusuzumaga
	Ese bagusuzumye kangahe uhageze?	-
	Ese ibise byari bimeze gute?	- Witaweho ute
	Ese gusunika byagenze gute?	- Igihe<br>- Ubufasha<br>- Imyifatire y'umusuzumyi<br>- Ubu babare
	Ese inda ya kabiri ya byajwe ite?	- Ibitekerezo

Ingingo	Ikibazo	Imfasha mibarize
	Wabonye umwana wawe nyuma y’igihe kingana gute umaze kubyara?	- Wamubonye bwangu<br>- Wahise wonsa<br>- Wabwiwe igitsina ryari
	Ese umwana y’igeze yitabwaho bi kwiye?	- Yakorewe iki<br>- Imyenda yumwana<br>- Waranyuzwe
	Hari imigenzo ikorwa nyuma yokubyara bya Kinyarwanda, iyo mihango waba uyizi? Niba uyizi, waba warifuje kuyikora ukagira inzitizi?	- Sobanura
	Mbwira uko wafashwe umaze kubyara utegereje gusezererwa?	- Kangahe<br>- Kugabanya ububabare<br>- Izsuzumwa ry'umwana
	Ese waba warahawe inyigisho mbere y’uko utaha? Nizihe? Zigira kamaro ki?	- Ayahe makuru<br>- Byarafashije
Murakoze kugihe cyanyu mumpaye no ku makuru. Muramutse mufite ibindi bibazo mwambaza kandi nti biza babuze kumpamagara. Hari ikindi mwifuza kumbwira? Mwambona kuri iyi numero?		
