# Supplementary material for: Listening to their voices: understanding rural women’s perceptions of good delivery care at the Mibilizi District Hospital in Rwanda
Source: BMC Womens Health. 2018 Feb 12;18:38. doi: 10.1186/s12905-018-0530-3 (PMC5809806; doi:10.1186/s12905-018-0530-3)
Supplement: Supplementary file 3 — Midwive’s interview guide. (PDF 67 kb) [file 12905_2018_530_MOESM3_ESM.pdf]

MIDWIFE INTERVIEW GUIDE

Interviewer	Name:	Date:	Location:
Interviewee	Name:	Sex	
	Position:		

Questions

1. Please take me through your typical working day.
   Probe:
   - Hours of duty per midwife per week
   - Number of night shifts per midwife
   - Annual leave respected
   - Average number of deliveries per day
   - Workload: some women are unattended to for this reason
   - How often do you have to monitor a woman during her active phase of labor?
   - How do you manage the third stage of labor here?

2. As a birth attendant, how do you feel about the services you provide?

   Probe:
   - Dissatisfaction/satisfaction; when and why it happens

3. As a birth attendant, what may be the potential obstacles for you to provide good delivery care?

   Probes:
   - Inadequate essential drugs in case of complications and emergencies
   - insufficient and inappropriate labor and postnatal rooms
   - Inadequate monitoring equipment e.g Foetoscope, Cardiotocogram (CTG)
   - Availability of drugs for pain management
   - Presence of oxygen and neonatal resuscitation equipment
   - Presence of incubators
   - Availability and access to blood transfusions
   - Presence of ambulance and prompt referral system
   - Client personality (easy/ difficult clients)
   - Workmate relation

4. How accessible is your hospital for the women in your catchment area?

   Probes:
   - Distance
   - Transportation from home to facility
   - Terrain/ weather

5. What is your ability to ensure maximum cleanliness, hygiene and respect aseptic procedures?
   Probes:
   - Constant water flow
   - Enough cleaners
   - How common are post-operation/puerperal infections
   - Adequate disinfecting material

6. What is your opinion on the cost of services you provide in regard to the socioeconomic status the women?
   Probes:
   - Cost of services
   - Subsidies
   - Confiscation of mother or property in case of failure to pay

7. What is your opinion on birth companions?
   Probe:
   - Are they helpful?
   - Choice of companion
   - Effect of husband's presence

8. How do you consider and adapt to cultural/traditional practices or beliefs?
   Probes:
   - Traditional birth rituals : facility adapted
   - Choice of sex of provider

9. Do women have to wait to be attended to after arrival at hospital?
   - On average how long does she have to wait?

10. How long does it take before the mother sees her newborn after delivery?

    - When do you proclaim the sex of the baby to her?

11. What do you consider prime after delivering the baby?
    - Mother/baby
    - Who do you give more attention to?

	12. What does your post delivery care include?<br>- How often are post delivery ward rounds done?<br><br>13. Any further care that you provide to mother after she delivers?<br><br>14. Do you provide information to mothers upon discharge?<br>Probes:<br>- What information?<br>- Mother and baby care<br>- Postnatal visits/ Immunization
Closing	I thank you for sharing experience with me and giving me your precious time. Are there any questions or further information you would like to share with me?<br>May I contact you in case I need further information? In case you want to contact me please don’t hesitate: Tel…
