# Supplementary material for: Listening to their voices: understanding rural women’s perceptions of good delivery care at the Mibilizi District Hospital in Rwanda
Source: BMC Womens Health. 2018 Feb 12;18:38. doi: 10.1186/s12905-018-0530-3 (PMC5809806; doi:10.1186/s12905-018-0530-3)
Supplement: Supplementary file 4 — Coding scheme (illustrating interview text analysis). (PDF 82 kb) [file 12905_2018_530_MOESM4_ESM.pdf]

# CODING SCHEME

**Listening to their voices: understanding rural women's perceptions on good delivery care at Mibilizi District Hospital in Rwanda.**

**Women's Interview Text Analysis**

**N.B:** In bold are themes, underlined are categories while the rest are sub-categories.

1. **Accessibility of Health Facility**
    - 1.1 Distance
        - 1.1.1 Landscape
        - 1.1.2 Kilometers from home to health facility
        - 1.1.3 Time of travel
        - 1.1.4 Geographical features (mountains, valleys, water bodies)
    - 1.2 Transport
        - 1.2.1 Car
        - 1.2.2 Foot
        - 1.2.3 Ingobyi (local stretcher)
        - 1.2.4 Motorcycle
        - 1.2.5 Bicycle
        - 1.2.6 Cost of transport
2. **Decision to Seek Care**
    - 2.1 Family influence
        - 2.1.1 Husband's decision/ opinion
        - 2.1.2 Mother in-law's decision/opinion
        - 2.1.3 Woman's decision
    - 2.2 Previous experience at hospital
        - 2.2.1 Previous negative birth outcome
        - 2.2.2 Previous positive birth outcome
        - 2.2.3 Complications during delivery
        - 2.2.4 Cesarean section/ Simple vaginal delivery
        - 2.2.5 Embarrassment
    - 2.3 Social factors
        - 2.3.1 Traditional birth attendants
        - 2.3.2 Sorcery/ witchcraft
        - 2.3.3 Birth rituals; placental burial, infibulations etcetera.
        - 2.3.4 Custom/ tradition
        - 2.3.5 Societal judgment
    - 2.4 Financial factors
        - 2.4.1 Source of income
        - 2.4.2 Sell of property
        - 2.4.3 Borrowing money
        - 2.4.4 Cost of services
3. **Health provider communication**
    - 3.1 Empathy
        - 3.1.1 Pity
        - 3.1.2 Warm welcome
        - 3.1.3 Reassuring language
        - 3.1.4 Polite language
        - 3.1.5 Explaining procedures
        - 3.1.6 Introducing oneself
        - 3.1.7 Waiting time for services
    - 3.2 Apathy
        - 3.2.1 Insulting/ harsh language
        - 3.2.2 Blame
        - 3.2.3 Negligence

3.2.4 Silence
3.2.5 Physical assault

**4. Emotional support**

4.1 Negative Feelings
4.1.1 Loneliness
4.1.2 Abandonment
4.1.3 Distress
4.1.4 Regret
4.1.5 Death

4.2 Positive Feelings
4.2.1 Supported
4.2.2 Self- esteem
4.2.3 Important
4.2.4 Courage
4.2.5 Love

4.3 Family Presence
4.3.1 Husband
4.3.2 Mother in-law
4.3.3 Relative or friend

**5. Privacy**

5.1 Medical procedure
5.1.1 Frequency of vaginal examinations
5.1.2 Number of persons doing vaginal exam
5.1.3 Explanation of procedure and use of exam before application
5.1.4 Manner of patient preparation for and transport to theater

5.2 Seclusion
5.2.1 Screening / Shielding from third persons
5.2.2 Presence of male attendants
5.2.3 Appropriateness of labor attire (hospital scrubs)

**6. Cost of services**

6.1 Mode of payment
6.1.1 Mutuelle de santé
6.1.2 Out-of- pocket
6.1.3 Amount paid

6.2 Difficulty in payment
6.2.1 Sell of property
6.2.2 Acquisition of loan
6.2.3 Payment exemption
6.2.4 Confiscation due to failure to pay

**7. Hygiene and cleanliness at facility**

7.1 Labor room
7.1.1 Delivery table clean and has protection
7.1.2 Use of gloves and disinfection material during procedures
7.1.3 Disposal of products of conception (POCs)
7.1.4 Smell of labor room

7.2 Admission room
7.2.1 Cleanliness of linen
7.2.2 Proximity of beds and number of women per bed
7.2.3 Cleanliness of bathroom and toilets
7.2.4 Availability of water
7.2.5 Availability of place to wash utensils

**8. Birth Experience**

8.1 Feeling
8.1.1 Joy
8.1.2 Fear
8.1.3 Regret
8.1.4 Pain

8.1.5 Death

**Midwives' Interview Text Analysis**

1. **Workload**
   1.1 Number of delivery on average per day
   1.2 Annual leave
   1.3 Attending medical conferences/seminar for CME
   1.4 Number of night shifts
   1.5 Number of working hours per day
2. **Promptness of care**
   2.1 Waiting time
   2.2 Response to emergencies
   2.3 Availability of drugs, supplies and equipment
   2.4 Adequacy of ambulances
   2.5 Adequacy of staff per duty
3. **Availability of infrastructure and material**
   3.1 Adequacy of admission beds
   3.2 Adequacy of delivery beds and rooms
   3.3 Adequacy of theaters
4. **Birth companion**
   4.1 Psychological support
   4.2 Physical support
   4.3 Consent for medical procedure
5. **Cost of care**
   5.1 Amount paid
   5.2 Exemption
   5.3 Mutuelle de santé
   5.4 Confiscation of patients
   5.5 Perceived effect of cost to quality of care
6. **Access to health facility**
   6.1 Distance
   6.2 Means Transport
   6.3 Landscape
   6.4 Availability of ambulance
7. **Information impartation**
   7.1 Information during labor
      7.1.1 Progress of cervical dilatation
      7.1.2 Fetal presentation and viability
   7.2 Information during the postpartum period
      7.2.1 Breastfeeding
      7.2.2 Personal and newborn's hygiene
      7.2.3 Nutrition
      7.2.4 Wound care
      7.2.5 Family planning
      7.2.6 Vaccination
8. **Patient compassion**
   8.1 Patient discrimination
   8.2 Promptness of care
   8.3 Communication
   8.4 Sympathy
